# Therapeutic efficacy of TBC3711 in monocrotaline-induced pulmonary hypertension

**DOI:** 10.1186/1465-9921-12-87

**Published:** 2011-06-23

**Authors:** Djuro Kosanovic, Baktybek Kojonazarov, Himal Luitel, Bhola K Dahal, Akylbek Sydykov, Teodora Cornitescu, Wiebke Janssen, Ralf P Brandes, Neil Davie, Hossein A Ghofrani, Norbert Weissmann, Friedrich Grimminger, Werner Seeger, Ralph T Schermuly

**Affiliations:** 1 University of Giessen Lung Center, Giessen, Germany; 2 Max-Planck-Institute for Heart and Lung Research, Bad Nauheim, Germany; 3 Institute for Cardiovascular Physiology, J.W. Goethe University, Frankfurt, Germany; 4 Pfizer Global Research and Development, Sandwich, UK

## Abstract

**Background:**

Endothelin-1 signalling plays an important role in pathogenesis of pulmonary hypertension. Although different endothelin-A receptor antagonists are developed, a novel therapeutic option to cure the disease is still needed. This study aims to investigate the therapeutic efficacy of the selective endothelin-A receptor antagonist TBC3711 in monocrotaline-induced pulmonary hypertension in rats.

**Methods:**

Monocrotaline-injected male Sprague-Dawley rats were randomized and treated orally from day 21 to 35 either with TBC3711 (Dose: 30 mg/kg body weight/day) or placebo. Echocardiographic measurements of different hemodynamic and right-heart hypertrophy parameters were performed. After day 35, rats were sacrificed for invasive hemodynamic and right-heart hypertrophy measurements. Additionally, histologic assessment of pulmonary vascular and right-heart remodelling was performed.

**Results:**

The novel endothelin-A receptor antagonist TBC3711 significantly attenuated monocrotaline-induced pulmonary hypertension, as evident from improved hemodynamics and right-heart hypertrophy in comparison with placebo group. In addition, muscularization and medial wall thickness of distal pulmonary vessels were ameliorated. The histologic evaluation of the right ventricle showed a significant reduction in fibrosis and cardiomyocyte size, suggesting an improvement in right-heart remodelling.

**Conclusion:**

The results of this study suggest that the selective endothelin-A receptor antagonist TBC3711 demonstrates therapeutic benefit in rats with established pulmonary hypertension, thus representing a useful therapeutic approach for treatment of pulmonary hypertension.

## Background

Pulmonary hypertension (PH) is a chronic life-threatening disease characterized by a progressive augmentation of pulmonary arterial pressure that finally leads to right ventricle failure and death. PH has a multicomplex pathology that includes a combination of pulmonary vascular remodelling, vasoconstriction and *in situ *thrombosis. The progressive pulmonary vascular remodelling is the attribute of PH pathology and is characterized by abnormalities of vascular cells, such as increased proliferation, migration and resistance to apoptosis [1,2]. Although the PH pathology is the subject of intensive research, the precise molecular mechanisms are not fully understood and successful therapeutic strategy to cure the disease is still needed.

An accumulating body of literature clearly underlines the central role of endothelial dysfunction in the development and progression of PH [3-5]. Endothelin (ET)-1 is synthesized by endothelial cells in the human vasculature and causes a strong and potent vasoconstriction [6,7]. ET-1 is primarily produced by endothelial cells and manifests effects through 2 G-protein-coupled receptors ET-A and ET-B. These receptors have a different localization and therefore cause the different biological responses. The ET-A receptors are mostly expressed on pulmonary artery smooth muscle cells (PASMCs), cardiomyocytes and fibroblasts, whereas the ET-B receptors are presented on endothelial cells and, to a lesser extent, on PASMCs [8]. After activation by ET-1, both receptor types located on PASMCs cause a potent vasoconstriction and proliferation of PASMCs [9]. The ET-B receptors expressed on endothelial cells mediate a vasodilatation through nitric oxide and cyclic guanosine monophosphate and prostacyclin production and ET-B receptor-mediated ET-1 clearance [10,11]. Additionally, it is shown that deficiency of the ET-B receptor markedly accelerates the progression of PH in monocrotaline (MCT)-injected rats [12]. Nishida *et al *suggest that ET-A receptor mediated action is exclusively involved in the pathogenesis of MCT-induced PH, although they could not rule out a protective role of ET-B receptor mediated actions [13]. These facts created a novel paradigm that selective ET-A receptor antagonism is more favorable than a nonselective ET-A/ET-B approach.

The right-heart failure is the final stage in progression of PH, and it is known that ET receptors are expressed on cardiomyocytes as well [14]. ET-1 causes cardiac hypertrophy [15,16], and it was shown that treatment with an ET-A receptor antagonist improved the hemodynamics and survival in rats with chronic heart failure [17]. More importantly, the selective ET-A receptor antagonists, such as LU135252, PD155080, BQ-123, BMS-193884, significantly reduced right-heart hypertrophy and improved heart function in the MCT model of PH [16,18-20].

Through the years many selective ET-A receptor antagonists, such as BQ-123 [16,21,22], YM598 [23], GF063 [24] and sitaxentan, were developed and exhibited beneficial therapeutic effects in experimental models of PH. Sitaxentan, a very potent and selective ET-A receptor antagonist, successfully prevented and reversed pulmonary vascular remodelling and right-heart hypertrophy in rat hypoxic model, whereas only the preventive effects in the MCT model of PH were observed [25,26].

A novel and highly potent ET-A receptor antagonist, TBC3711 (IC_50 _= 0.08 nM) has been reported [27-30]. This compound shows a significantly stronger ET-A/ET-B selectivity (441.000-fold) as compared with sitaxentan (6.500-fold) and thus can represent a novel specific and selective therapeutic approach for the treatment of PH. This study explored, for the first time to our knowledge, the therapeutic efficacy of TBC3711 in a well-established MCT model of pulmonary arterial hypertension in rats and the effects of TBC3711 on (1) hemodynamics, (2) pulmonary vascular remodelling and (3) right ventricular (RV) hypertrophy and remodelling on MCT-induced pulmonary hypertension in rats.

## Methods

### Experimental design

Adult male Sprague-Dawley rats (300-350 g body weight (BW)) were given a subcutaneous injection of saline (healthy control, n = 8) or MCT (60 mg/kg BW, n = 30). MCT-injected rats were randomized into 2 groups and were treated orally by gavage from 21 to 35 days either with TBC3711 (MCT-TBC3711 group, 30 mg/kg BW/day, n = 16) or placebo (MCT-placebo group, methyl-cellulose, n = 14). The BW changes and survival were monitored from 21 to 35 days for all experimental groups (Additional file 1, Figure S1). All animal studies were performed according to the guidelines of the University of Giessen and were approved by the local authorities.

### Echocardiographic measurements

Echocardiographic measurements of pulmonary artery acceleration time (ACT), tricuspid annular plane systolic excursion (TAPSE), cardiac output (CO), heart rate (HR), right ventricular dimensions (RVD) and right ventricular wall thickness (RVWT) were performed for all experimental groups. Anesthesia was induced with 3% isoflurane gas and maintained with 1.0% to 1.5% isoflurane in room air supplemented with 100% O_2_. Rats were laid supine on a heating platform with all legs taped to electrocardiogram electrodes for monitoring heart rate (HR). Body temperature was monitored via a rectal thermometer (Indus Instruments, Houston, TX, USA) and maintained at 36.5°C to 37.5°C using a heating pad and lamp. The rat's chest was shaved and treated with a chemical hair remover to reduce ultrasound attenuation. To provide a coupling medium for the transducer, a warm ultrasound gel was spread over the chest wall. Transthoracic 2-dimensional, M-mode and Doppler imaging were performed with a high-resolution imaging system equipped with a 25-MHz transducer (VisualSonics, Toronto, ON, Canada). Right ventricular wall thickness (RVWT) was measured in the modified parasternal long-axis view. Right ventricular dimension (RVD) was measured from the right ventricle (RV) outflow tract view at the level of the aortic valve. For determination of tricuspid annular plane systolic excursion (TAPSE), M-mode cursor was oriented to the junction of the tricuspid valve plane with the RV free wall using the apical 4-chamber view. Pulmonary artery acceleration time (ACT) (Additional file 2, Figure S2) was measured from the pulsed-wave Doppler flow velocity profile of the RV outflow tract in the parasternal short-axis view and defined as the interval from the onset to the maximal velocity of forward flow [31,32]. Cardiac output (CO) was calculated as the product of the velocity time integral of the pulsed-Doppler tracing in the left ventricle (LV) outflow tract, the cross-sectional area of the LV outflow tract and the HR [33]. All echocardiographic parameters were calculated offline using tool section of the VisualSonics Vevo770 System. All the studies were performed by an experienced sonographer who was blinded to results of invasive and morphometric studies.

### Invasive hemodynamic measurement of the right ventricular systolic pressure (RVSP) and systemic arterial pressure (SAP)

The animals were initially anesthetized intraperitoneally with a mixture of ketamine (50 mg/kg) and medetomidin (100 μg/kg) for invasive hemodynamic measurements. A right-heart catheter was inserted through the right jugular vein for measurement of right ventricular systolic pressure (RVSP) and the left carotid artery cannulation was performed for systemic arterial pressure (SAP) measurement, as described previously [34-36]. The partial arterial oxygen pressure (paO_2_) was measured by blood gas analysis for the determination of oxygenation index (paO_2_/FiO_2_) [36].

### Assessment of right ventricular (RV) hypertrophy and paraffin embedding of the hearts

The RV wall was separated from the left ventricular (LV) wall and ventricular septum (S). RV hypertrophy was expressed as the wet weight ratio of the RV wall and free LV wall with ventricular septum (RV/(LV+S)), as already described [34-36]. RVWT was measured by using a standard micrometer calliper (RVWT invasive). At the end of all heart hypertrophy measurements the RV wall, LV wall and S were fixed with formalin (3.5%-3.7%) and after dehydration were embedded in paraffin for histologic analysis.

### Morphometric analysis of the lung vessels

The left lungs from all experimental groups were formalin-fixed and paraffin embedded for the morphometric analysis of the pulmonary vessels (medial wall thickness and degree of muscularization) and for the assessment of index of proliferation (IOP), as described previously [35].

### Determination of collagen content and cardiomyocyte size in right ventricles

Freshly dissected RV tissues were fixed in 3.5% to 3.7% formalin solution overnight, then dehydrated and embedded in paraffin and sectioned at a thickness of 3 μm. To detect collagen fibres, the RV sections were stained with 0.1% Sirius red F3B (Niepoetter, Bürstadt, Germany) in picric acid (Fluka, Buchs, Germany). Photomicrographs were quantified to determine the interstitial collagen fraction by using Leica QWin V3 computer-assisted image analysis software (Leica Microsystem, Wetzlar, Germany). Average data reflect results from at least 4 different hearts in each group, as described previously [37]. For cardiomyocyte size determination the transverse section of formalin-fixed paraffin-embedded RVs were stained with fluorescein isothiocyanate-conjugated wheat germ agglutinin (Sigma Aldrich, Steinheim, Germany). Nuclei were stained with diamidino phenylindole (Invitrogen, Darmstadt, Germany) and mounted with fluorescent mounting medium (Dako, Hamburg, Germany). The cross-sectional area per cardiomyocyte was measured by using Leica QWin software [38].

### Data analysis

All data were expressed as mean ± SEM. The different experimental groups were statistically analyzed by 1-way ANOVA and Newman-Keuls post hoc test for multiple comparisons. *P *values of < 0.05 were considered as statistically significant. The correlations between different invasive and echocardiographic parameters were analyzed using a Spearman analysis.

## Results

### Effect of TBC3711 on hemodynamics in monocrotaline-induced pulmonary hypertension

**Figure 1:**
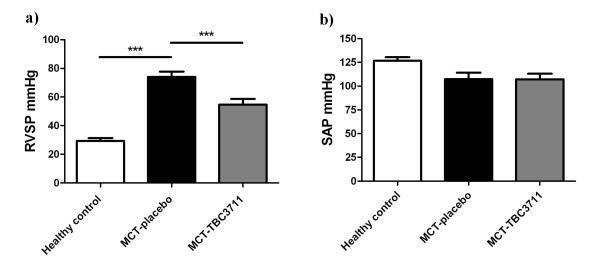
**Effect of TBC3711 on hemodynamics in monocrotaline (MCT)-induced pulmonary hypertension**. Rats were treated with TBC3711 (n = 14) or vehicle (n = 9) from day 21-35 after MCT-injection. TBC3711 (MCT-TBC3711, 30 mg/kg body weight/day) was administered once per day orally by gavage. Equal volume of the vehicle (methyl-cellulose) was given to a placebo group (MCT-placebo). After 35 days all animals were humanely killed for hemodynamic measurements. (a) Right ventricular systolic pressure (RVSP) and (b) Systemic arterial pressure (SAP) are given. Bars represent mean ± SEM. One-way ANOVA with Newman-Keuls multiple comparison post-hoc test was performed for statistical analysis. ***P < 0.001.

MCT induced a robust and severe PH in rats as reflected by a significantly increased right ventricular systolic pressure (RVSP) in the placebo group (74.0 ± 3.6 vs 29.3 ± 1.9 mmHg in healthy group; Figure 1a). Oral treatment with TBC3711 showed a significant decrease in RVSP compared with placebo (54.7 ± 3.8 vs 74.0 ± 3.6 mmHg). SAP was not changed in any of experimental groups (Figure 1b).

### Effect of TBC3711 on pulmonary artery acceleration time, cardiac output, total pulmonary resistance and oxygenation index

**Figure 2:**
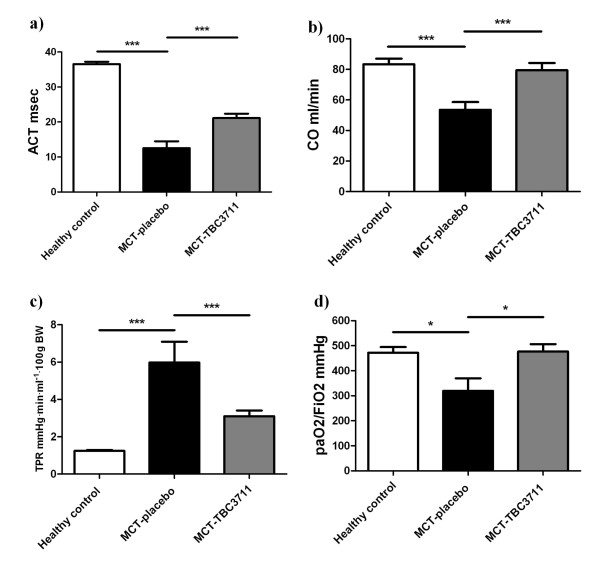
**Effect of TBC3711 on pulmonary artery acceleration time (ACT), cardiac output (CO), total pulmonary resistance (TPR) and oxygenation index (paO_2_/FiO_2_) in monocrotaline (MCT)-induced pulmonary hypertension**. Rats were treated with TBC3711 (30 mg/kg body weight/day, n = 14) or vehicle (n = 9) from day 21-35 after MCT-injection accompanied by echocardiography measurement and blood gas analysis. (a) Pulmonary artery acceleration time (ACT), (b) Cardiac output (CO), (c) Total pulmonary resistance (TPR) and (d) oxygenation index (paO_2_/FiO_2_) of different experimental groups are given. Bars represent mean ± SEM. One-way ANOVA with Newman-Keuls multiple comparison post-hoc test was performed for statistical analysis. *P < 0.05, ***P < 0.001.

Pulmonary artery acceleration time (ACT) was measured as described in Methods by using echocardiography (Additional file 2, Figure S2). MCT induced a severe PH in rats, which is reflected by a significant reduction in ACT compared with healthy animals (Figure 2a). Animals treated with TBC3711 showed a significant increase of ACT in comparison with placebo. CO was decreased in MCT-placebo rats as compared with healthy controls (53.5 ± 4.8 vs 83.4 ± 3.5 ml/min). TBC3711 caused a significant increase of CO compared with the placebo group (Figure 2b). Total pulmonary resistance (TPR) was defined as RVSP divided by cardiac index (CI) and was significantly augmented in the placebo group compared with healthy rats (5.98 ± 1.03 vs 1.24 ± 0.04 mmHg × min × ml-¹ × 100 g BW) and TBC3711 effectively reduced the value of TPR (3.10 ± 0.29 vs 5.98 ± 1.03 mmHg × min × ml-¹ × 100 g BW in placebo group; Figure 2c). The oxygenation index (paO_2_/FiO_2_) was significantly reduced in the MCT-placebo group compared with healthy animals (320 ± 46 vs 472 ± 20 mmHg). The treatment with TBC3711 improved oxygenation index compared with the placebo group (477 ± 28 vs 320 ± 46 mmHg) (Figure 2d).

### TBC3711 reduced right ventricular hypertrophy and improved right-heart function

**Figure 3:**
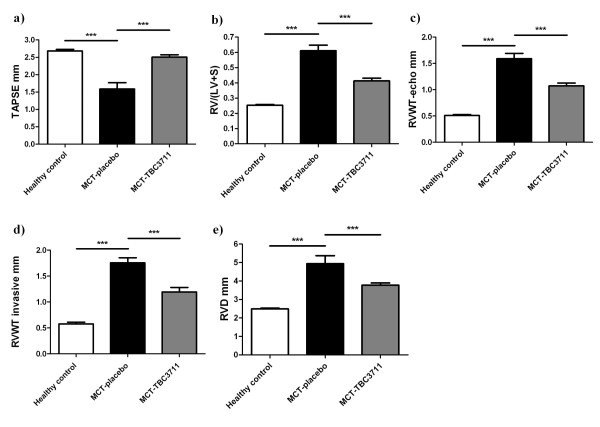
**TBC3711 reduced a right ventricular hypertrophy and improved a right-heart function in monocrotaline model of pulmonary hypertension**. Tricuspid annular plane systolic excursion (TAPSE) was measured by echocardiography. The right ventricle (RV) wall was separated from the left ventricle (LV) wall and ventricular septum (S). RV hypertrophy (heart ratio) was expressed as the weight ratio of the RV wall and free LV wall with ventricular septum (RV/(LV+S)) in different experimental groups. Additionally, right ventricular wall thickness (RVWT) and right ventricular dimensions (RVD) were measured by echocardiography. (a) TAPSE, (b) RV/(LV+S), (c) RVWT measured by echocardiography (RVWT-echo), (d) RVWT measured by invasive approach (RVWT invasive) and (e) RVD measured by echocardiography are shown for all experimental groups. Bars represent mean ± SEM. One-way ANOVA with Newman-Keuls multiple comparison post-hoc test was performed for statistical analysis. ***P < 0.001.

Tricuspid annular plane systolic excursion (TAPSE), as a measure of RV systolic function was significantly decreased in the placebo group (1.59 ± 0.14 vs 2.68 ± 0.04 mm in healthy animals; Figure 3a). TBC3711 remarkably improved TAPSE as compared with placebo (2.51 ± 0.06 vs 1.59 ± 0.14 mm). The RV hypertrophy was evident from a significantly increased RV/(LV+S) ratio of MCT-injected rats receiving placebo, compared with the healthy rats (0.61 ± 0.03 vs 0.25 ± 0.01; Figure 3b). Treatment with TBC3711 significantly reduced the RV/(LV+S) ratio in comparison with placebo (0.41 ± 0.02 vs 0.61 ± 0.03). RVWT was significantly increased in MCT-injected rats as measured by both invasive and echocardiographic approaches. The oral treatment of rats with TBC3711 showed a significantly reduced RVWT compared with placebo group (Figures 3c, d). RVD were augmented in rats injected with MCT (4.94 ± 0.41 vs 2.49 ± 0.04 mm in healthy controls) and chronic treatment with TBC3711 caused the significant decrease of RVD compared with placebo group (3.78 ± 0.11 vs 4.94 ± 0.41 mm; Figure 3e).

### The correlation between different parameters measured by invasive and echocardiographic approaches in monocrotaline-injected rats

**Figure 4:**
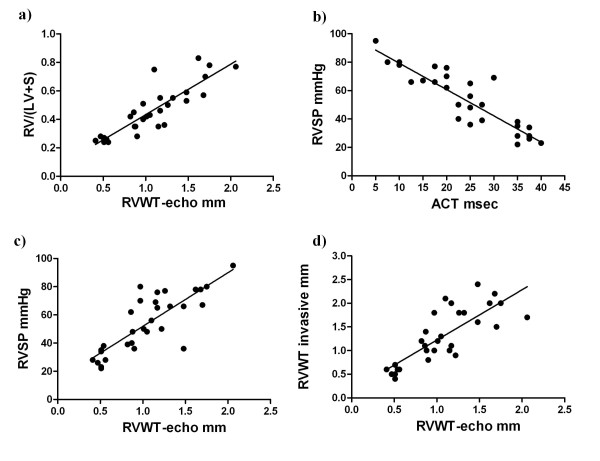
**The correlation between different parameters measured by invasive and echocardiographic approaches in monocrotaline (MCT)-injected rats**. Correlations between invasive and echocardiographic measurements of different parameters are shown. (a) weight ratio of the right ventricle (RV) wall and free left ventricle (LV) wall with ventricular septum (RV/(LV+S)) and right ventricular wall thickness measured by echocardiography (RVWT- echo), (b) right ventricular systolic pressure (RVSP) and pulmonary artery acceleration time (ACT), (c) RVSP and RVWT-echo and (d) RVWT invasive and RVWT-echo. The Spearman analysis was performed for statistical analysis. All *P *< 0.0001.

The Spearman analysis demonstrated a significant correlation between invasive measurements of different parameters and echocardiographic (echo) measurements (Figure 4). RV/(LV+S) and RVWT-echo (r = 0.88, *P *< 0.0001); RVSP and RVWT-echo (r = 0.81, *P *< 0.0001) and RVWT invasive and RVWT-echo (r = 0.81, *P *< 0.0001) showed the noticeable positive correlation. Here we also demonstrated that RVSP significantly negatively correlates with ACT (r = -0.89, *P *< 0.0001).

### Effect of TBC3711 on monocrotaline-induced pulmonary vascular remodelling

**Figure 5:**
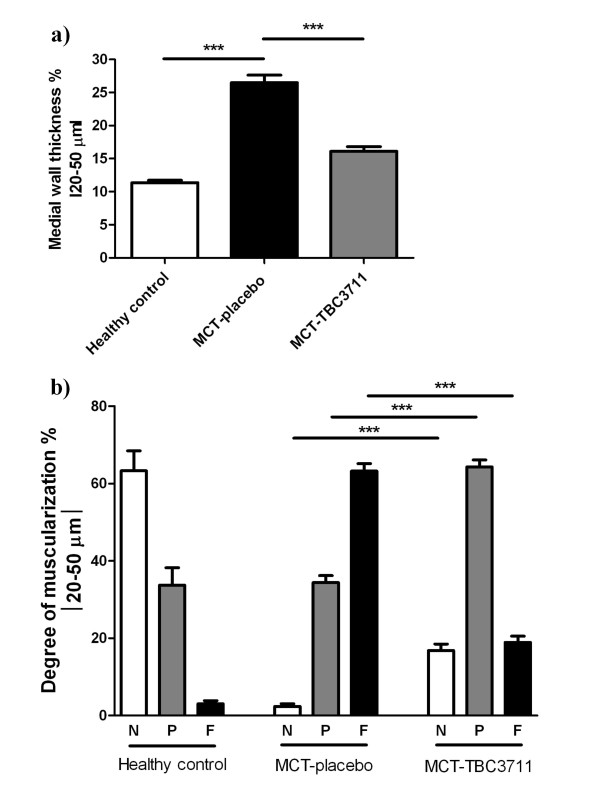
**Effect of TBC3711 on monocrotaline (MCT)-induced pulmonary vascular remodelling**. The rat lung sections were stained with elastica staining for medial wall thickness determination of pulmonary vessels in all experimental groups (a). The rat lung sections were also immunostained for α-smooth muscle actin and von Willebrand factor. (b) Proportion of non (N), partially (P) or fully (F) muscularized pulmonary vessels, as a percentage of total pulmonary vessel cross section (sized 20-50 μm) is given for different experimental groups. Bars represent mean ± SEM. One-way ANOVA with Newman-Keuls multiple comparison post-hoc test was performed for statistical analysis. ***P < 0.001.

To assess the effect of TBC3711 on pulmonary vascular remodelling, we analyzed medial wall thickness (Figure 5a; Additional file 3, Figure S3a) and degree of muscularization (Figure 5b; Additional file 3, Figure S3b) of peripheral pulmonary vessels (20-50 μm). MCT injection resulted in an enhanced pulmonary artery remodelling as evident from a significantly increased medial wall thickness in placebo group (26.5 ± 1.1 vs 11.3 ± 0.3% in healthy control). ET-A receptor antagonist TBC3711 showed the effective reduction of medial wall thickness in the treated group compared with placebo (16.1 ± 0.7 vs 26.5 ± 1.1%). MCT injection in rats resulted in augmented pulmonary arterial muscularization as evident from an increase in the number of fully muscularized arteries in placebo group (63.2 ± 1.8 vs 3.0 ± 0.8% in healthy controls). The number of nonmuscularized vessels was significantly reduced in MCT-placebo group as compared with healthy control (2.4 ± 0.6 vs 63.3 ± 4.7%). TBC3711 caused the marked reduction of pulmonary vascular remodelling as reflected by a significantly decreased fully muscularized vessels in treated group compared with placebo (18.9 ± 1.5 vs 63.2 ± 1.8%). Also, the number of partially muscularized arteries was significantly increased in TBC3711 treated group (64.3 ± 1.7 vs 34.4 ± 1.6% in placebo group).

### TBC3711 reduced the index of proliferation

**Figure 6:**
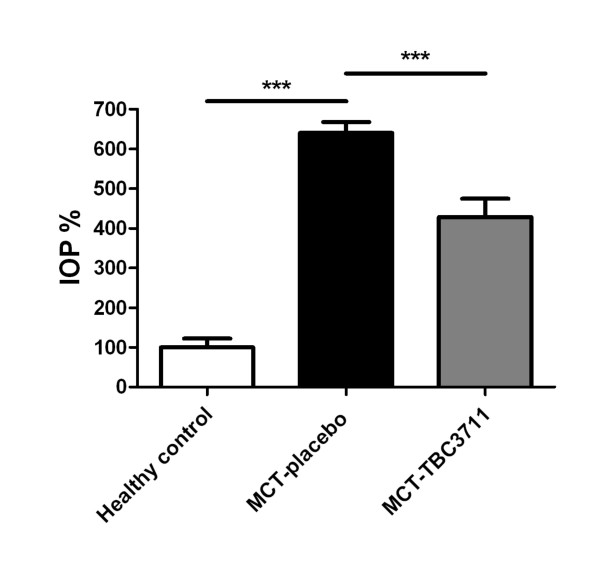
**TBC3711 reduced the index of proliferation (IOP) of perivascular cells in monocrotaline (MCT) model of pulmonary hypertension**. *In situ *proliferation of perivascular cells in rat lungs was investigating by using immunostaining of lung tissues with an anti-proliferating cell nuclear antigen rabbit polyclonal antibody, as explained in Methods. IOP of different experimental groups is shown. Bars represent mean ± SEM. One-way ANOVA with Newman-Keuls multiple comparison post-hoc test was performed for statistical analysis. ***P < 0.001.

MCT caused a strong *in situ *proliferation of perivascular cells (Additional file 3, Figure S3c) in the small pulmonary vessels (20-50 μm), as evident from increased IOP (Figure 6), as compared with healthy controls (640 ± 28 vs 100 ± 22%). TBC3711 showed a significant reduction in IOP, in comparison with placebo group (428 ± 46 vs 640 ± 28%).

### Effect of TBC3711 on collagen content and cardiomyocyte size in right ventricles of monocrotaline-injected rats

**Figure 7:**
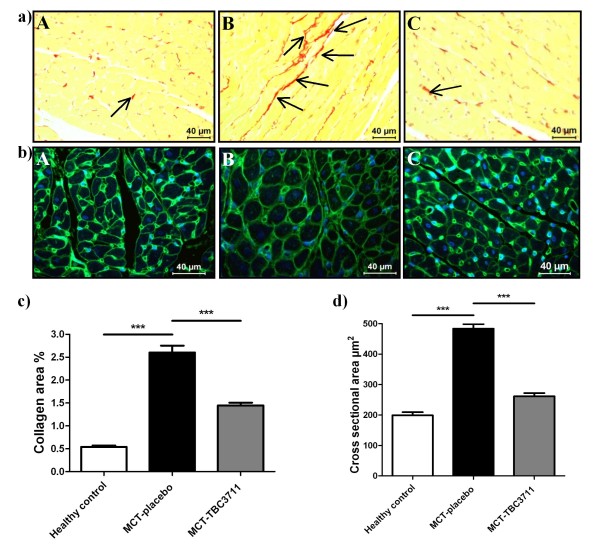
**Effect of TBC3711 on collagen content and cardiomyocyte size in right ventricles of monocrotaline (MCT)-injected rats**. Right ventricle (RV) sections were stained with 0.1% Sirius Red in picric acid to assess the collagen content and also with fluorescein isothiocyanate conjugated wheat germ agglutinin/diamidino phenylindole to assess the cardiomyocyte size. Photomicrographs were quantified to determine the interstitial collagen fraction and cardiomyocyte size by using computer-assisted image analysis software. (a, b) Representative photomicrographs are presented (A - healthy control, B - MCT-placebo and C - TBC3711 treated group; arrows indicate collagen formation - red color). (c) Collagen area and (d) cross sectional area are shown for different experimental groups. Bars represent mean ± SEM. One-way ANOVA with Newman-Keuls multiple comparison post-hoc test was performed for statistical analysis. ***P < 0.001.

Right ventricle collagen content (Figure 7a, c) was markedly increased in rats with MCT-induced pulmonary hypertension compared with healthy animals (2.60 ± 0.14 vs 0.54 ± 0.03%). TBC3711 significantly decreased the collagen area in comparison with placebo group (1.45 ± 0.05 vs 2.60 ± 0.14%). Cardiomyocytes hypertrophy (Figure 7b, d) measurement was expressed as cross-sectional area (μm^2^) per cardiomyocyte. MCT injection significantly increased a cardiomyocyte size (484 ± 12 vs 199 ± 8 μm^2 ^in healthy rats). TBC3711 noticeably reduced the cardiomyocyte size compared with placebo group (261 ± 9 vs 484 ± 12 μm^2^).

## Discussion

The main results from the present study are that (a) TBC3711 significantly improved hemodynamics in MCT-induced PH in rats and noticeable reduced pulmonary vascular remodelling and perivascular proliferating cells and (b) TBC3711 chronic treatment also strongly diminished right-heart remodelling as evident from collagen content and cardiomyocyte size, which was followed by a significantly decreased RV hypertrophy.

Over the years, many different therapeutic options have been investigated for the treatment of pulmonary hypertension, such as PDE5 inhibitors, prostacyclin analogs and endothelin-receptor antagonists [39,40]. Although these therapeutic approaches improved the quality of life and prolonged survival of patients with pulmonary hypertension, novel clinical options to achieve the ultimate goal of reversing the progressive pulmonary vascular remodelling and RV hypertrophy are needed.

Although it is not exactly clear what triggers the initiation of PH, a growing body of literature implicates the main role of ET system in the development and progression of the disease [4,5,41]. ET-1, an extremely strong vasoconstrictor primarily produced by endothelial cells, is significantly elevated in both human and MCT-induced PH [42,43]. ET-1 achieves the biological effects through 2 G protein-coupled receptor isoforms ET-A and ET-B. These receptors have a distinct localization, unique affinities and locations for binding ET-1 and therefore cause different biological responses [44]. The ET-A receptors are mostly expressed on PASMCs, cardiomyocytes and fibroblasts. When expressed on PASMCs, the ET-B receptors together with ET-A receptors mediate vasoconstriction and proliferation of vascular cells, but when expressed on endothelial cells the ET-B receptors cause vasodilatation and ET-1 clearance [9-11,44]. Ivy *et al *have shown that ET-B receptor deficiency accelerates the progression of PH in MCT rat model, and Nishida *et al *discussed their findings and suggested that ET-A receptor-mediated action is exclusively involved in the pathogenesis of MCT-induced PH, although they could not rule out a protective role of ET-B receptor-mediated actions [12,13]. All these findings pointed toward the requirement of a novel therapeutic approach in the direction of selective ET-A receptor antagonism, although nonselective approach (Bosentan) also showed the effective reduction of the PH in animal models [45,46].

Sitaxentan, a potent and highly selective ET-A receptor antagonist, prevented and reversed pulmonary vascular remodelling and cardiac hypertrophy in hypoxia models, whereas only the preventive effects were observed in MCT and over-circulation models of PH [3,25,26]. A series of clinical studies were performed with sitaxentan, named the Sitaxentan to Relieve Impaired Exercise Studies (STRIDE) [3,47]. Collectively, these studies showed the benefits of selective ET-A receptor antagonism in the setting of human forms of PH. Unfortunately, due to a pattern of idiosyncratic liver function abnormalities, sitaxentan was removed from the market in December 2010.

In the experiments outlined in the current manuscript, we used a novel, highly potent ET-A receptor antagonist (IC_50 _= 0.08 nM), named TBC3711. This follow-up compound was discovered following chemical substitution with variety of electron-withdrawing groups at the ortho position on the anilino ring [28]. TBC3711 shows a good oral bioavailability in rats and significantly stronger ET-A/ET-B selectivity (441.000-fold) as compared with sitaxentan (6500-fold) [27-29]. These advantages of TBC3711 indicate the need to investigate the therapeutic potency of this compound as a novel therapeutic option.

TBC3711 improved hemodynamics, reduced pulmonary vascular resistance and increased cardiac output in MCT rats treated with this compound in comparison with placebo group, as evident from both invasive and noninvasive measurements. Also, rats treated with TBC3711 had significantly higher arterial oxygenation compared with placebo group. Previously, it was shown that ACT inversely correlates with mean pulmonary arterial pressure [48-50]. In agreement with this finding we demonstrated that RVSP significantly negatively correlates with ACT, suggesting the importance of a noninvasive approach for prognostic significance in PH. Corroborating with hemodynamic data, TBC3711 significantly reduced pulmonary vascular remodelling as evident from decreased medial wall thickness and degree of muscularization of intra-acinar arteries (20-50 μm diameter). The decrease of pulmonary vascular remodelling may be associated with notably reduced level of vascular cell proliferation *in situ*, as evident from IOP. This is in agreement with literature that suggests the role of the ET system in the proliferation of vascular cells [3]. Additionally, MCT-injected rats treated with TBC3711 exhibited slightly improved survival and body weights compared with placebo (Additional file 1, Figure S1). Furthermore, no significant effect was observed on heart rate, suggesting that improvement in cardiac output after TBC3711 treatment was due to increased stroke volume (Additional file 4, Figure S4). Our findings that selective ET-A receptor antagonism in MCT-model of PH exhibited beneficial effects on hemodynamic changes and pulmonary vascular remodelling are in line with literature [21-23,51-53].

Brunner *et al *demonstrated that ET-A receptor antagonism alleviated cardiac dysfunction via improved Ca^2+ ^handling in MCT-induced RV hypertrophy, suggesting the role of ET-A receptors in RV dysfunction [18]. TBC3711 therapy significantly improved RV systolic function in the MCT-induced PH model, as evident from TAPSE measurement. TBC3711 strongly reduced the RV hypertrophy as evident from invasive measurements of heart ratio (RV/(LV+S)) and RVWT and echocardiographic measurements of RVWT and RV dimensions. Because remodelling of the right-heart structure is an attribute of the RV hypertrophy, we also investigated the effects of TBC3711 on cardiomyocytes hypertrophy and cardiac fibrosis and found that both parameters were significantly diminished in treated rats. The role of the ET system in pathogenesis of RV hypertrophy is not clear and very little is known. Jasmin *et al *have shown in the MCT model of PH that right ventricular ET-1 levels and ET-B receptor density were significantly increased, whereas ET-A receptors were not affected. Interestingly, they have shown that these alterations in the ET system in the RV were noticeably attenuated by treatment with selective ET-A receptor antagonist [19]. From the other side, Miyauchi *et al *in 1993 concluded that inhibition of RV hypertrophy by selective ET-A receptor antagonist BQ-123 in MCT-injected rats may also be because of the blockade of excessive stimulation of the heart by ET-1 in addition to the prevention of PH [16]. In 2000 Miyauchi *et al *found that there was a significant increase of atrial natriuretic peptide expression in the right-heart of MCT-injected rats, which is a marker for the failing heart and that increase was strongly prevented by selective ET-A receptor antagonism [20]. Whether the reduction of right-heart hypertrophy in our study was solely due to improved hemodynamics or to a contribution from direct effect of TBC3711 on the RV, was not deeply investigated here, and we do not have data to resolve this question.

In summary, we successfully demonstrated that daily oral treatment with TBC3711 in the dose of 30 mg/kg of BW/day impaired the progression of PH, as evident from significantly improved hemodynamics, RV hypertrophy and remodelling and pulmonary vascular remodelling in MCT-induced PH model in rats. Although some manifestations of human PH pathology, such as neointima formation and plexiform lesions are not covered by this model, other important pathological characteristics, such as initial endothelial injury, increased perivascular inflammation and *de novo *muscularization of small pulmonary arteries are shared features between human disease and MCT model. The animal models that more closely mimic human PH were reported in recent years, such as combination of MCT and one-sided pneumonectomy and hypoxia in combination with vascular endothelial growth factor receptor (VEGFR)-2 inhibitor SU5416. TBC3711 can thus be anticipated to yield therapeutic benefit in these animal models and can be used for further investigation. Additionally, TBC3711 can be used as a useful tool to further elucidate the role of ET system in recently found factors that are implicated in pulmonary vascular remodelling process, such as signal transducers and activators of transcription-3 (STAT3), nuclear factor of activated T cell (NFAT) and miR-204 [54,55]. Recently, Nagendran *et al *showed the putative contractile and metabolic effects of ET-1 antagonist on RV functions, which may have an important clinical value. Therefore, the potential future studies with TBC3711 may reveal if this antagonist has similar effects [56]. Previously it was shown that TBC3711 ameliorated the hypoxia-induced PH in the newborn piglet, suggesting the effectiveness of the compound in pulmonary hypertension caused by different stimulus, like hypoxia [27]. Taken together, the findings from our study indicate that TBC3711 with its beneficial properties, such as good oral bioavailability (~100% in rats), high potency (ET-A receptor IC_50 _= 0.08 nM) and strong selectivity (441.000-fold) [28] may offer a new therapeutic approach to cure PH.

## Competing interests

The authors declare that they have no competing interests.

## Authors' contributions

DK, HAG, NW, WS, FG and RTS conceived and designed the study. DK, BK and HL performed experiments. DK, BK, HL, BKD, HAG, NW, FG, WS and RTS analyzed and interpreted data. AS and TC were involved in interpretation of data. DK and RTS drafted and finalized the manuscript. BKD, AS, TC, WJ, RPB and ND were involved in revising the manuscript for important intellectual content. All authors read and approved the final manuscript.

## Supplementary Material

Additional file 1 **Figure S1. Effect of TBC3711 on body weight changes and survival in monocrotaline (MCT)-induced pulmonary hypertension**. Body weights and survival were monitored each day for last 2 weeks of experiments and the mean values of those 2 parameters on day 22 were considered to be 100%. (a) The survival curves and (b) body weight changes of different experimental groups and time points are shown. Click here for file

Additional file 2 **Figure S2. Representative photographs from echocardiography**. As described in Methods, the pulmonary artery acceleration time (ACT) was measured by echocardiography. The representative photographs from different experimental groups are shown (A - healthy control, B - monocrotaline (MCT)-placebo and C - MCT-TBC3711). Click here for file

Additional file 3 **Figure S3. Representative photomicrographs from elastica staining and immunostainings**. (a) elastica staining and (b) double immunostaining (anti-α-smooth muscle actin (purple/violet color) and anti-von Willebrand factor (brown color) antibodies) were performed to assess medial wall thickness and degree of muscularization, the 2 well-known measures of pulmonary vascular remodelling. Immunostaining with anti-proliferating cell nuclear antigen (PCNA) antibody (c) was performed to assess the *in situ *proliferation state of pulmonary vascular cells. Arrows indicate the PCNA-positive nuclei/cells. The representative photographs from stained lung tissues of different experimental groups are shown (A - healthy controls, B - monocrotaline (MCT)-placebo and C - MCT-TBC3711). Click here for file

Additional file 4 **Figure S4. Effect of TBC3711 on heart rate (HR)**. The heart rate (beats/min) was measured by echocardiography, as described in Methods and values of different experimental groups are given. Click here for file
