# Supplementary figures and images for: Therapeutic efficacy of TBC3711 in monocrotaline-induced pulmonary hypertension
Source: Respir Res. 2011 Jun 23;12(1):87. doi: 10.1186/1465-9921-12-87 (PMC3141422; doi:10.1186/1465-9921-12-87)

## Slide 1
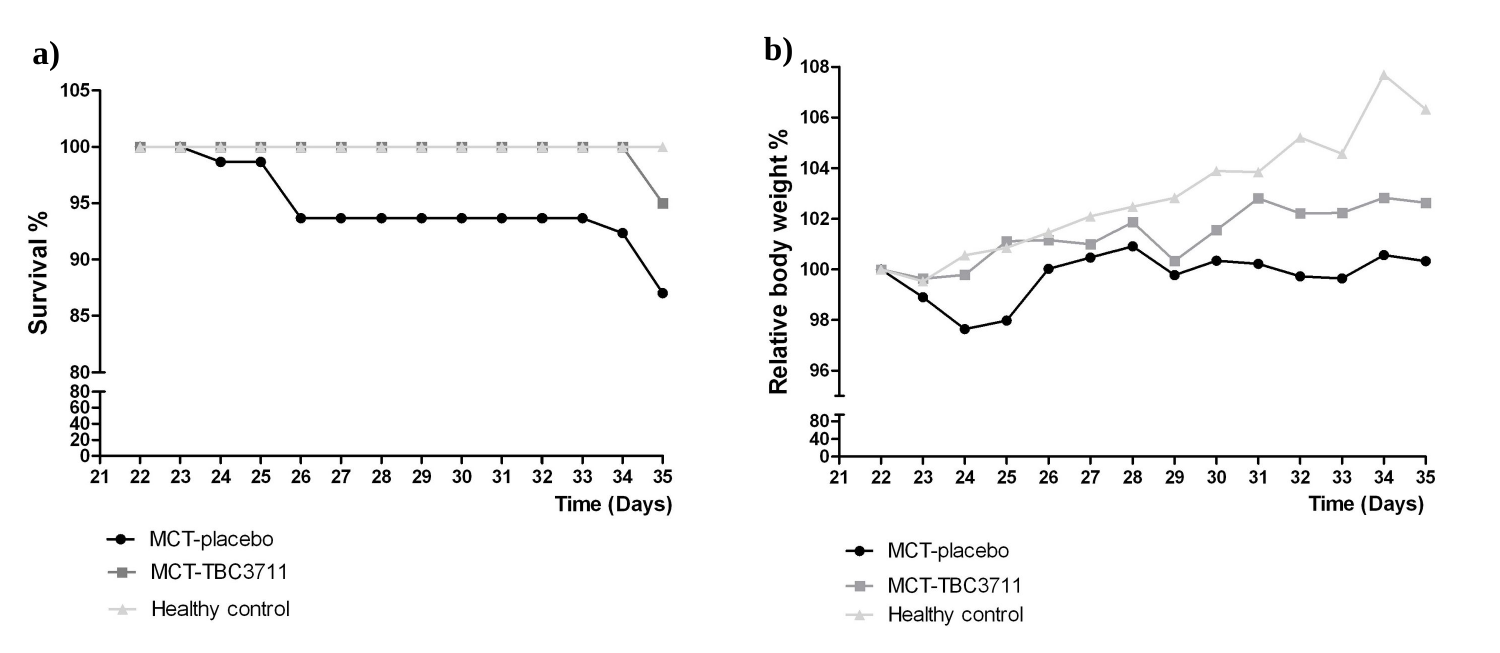

b)
a)

Supplement: Additional file 1 — Figure S1. Effect of TBC3711 on body weight changes and survival in monocrotaline (MCT)-induced pulmonary hypertension. Body weights and survival were monitored each day for last 2 weeks of experiments and the mean values of those 2 parameters on day 22 were considered to be 100%. (a) The survival curves and (b) body weight changes of different experimental groups and time points are shown. [file 1465-9921-12-87-S1.PPT]

## Slide 1
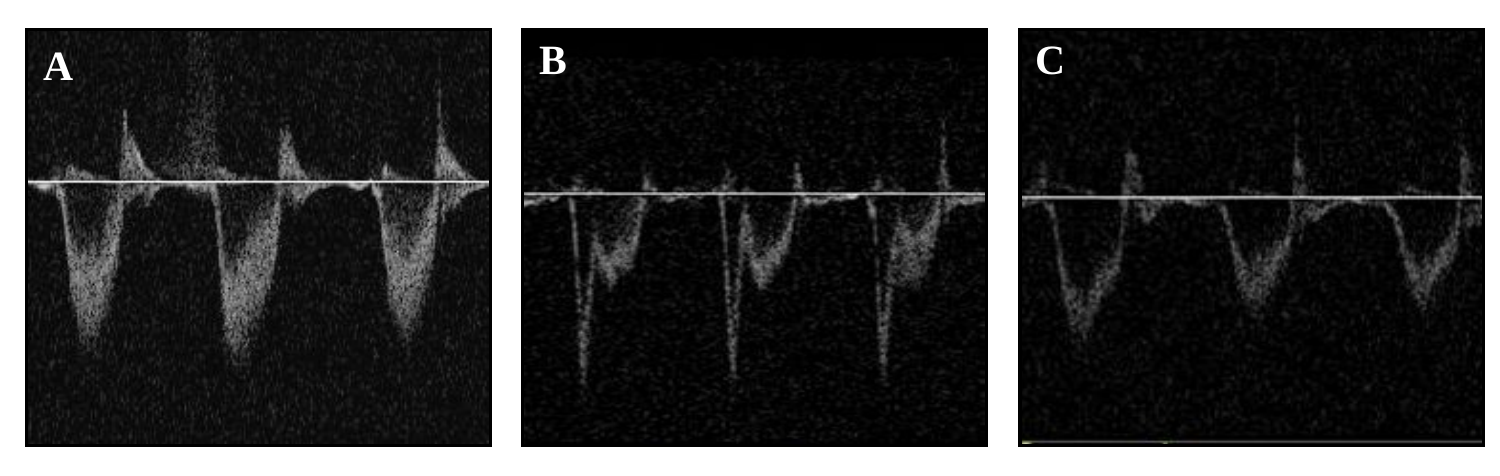

B
C
A
A

Supplement: Additional file 2 — Figure S2. Representative photographs from echocardiography. As described in Methods, the pulmonary artery acceleration time (ACT) was measured by echocardiography. The representative photographs from different experimental groups are shown (A - healthy control, B - monocrotaline (MCT)-placebo and C - MCT-TBC3711). [file 1465-9921-12-87-S2.PPT]

## Slide 1
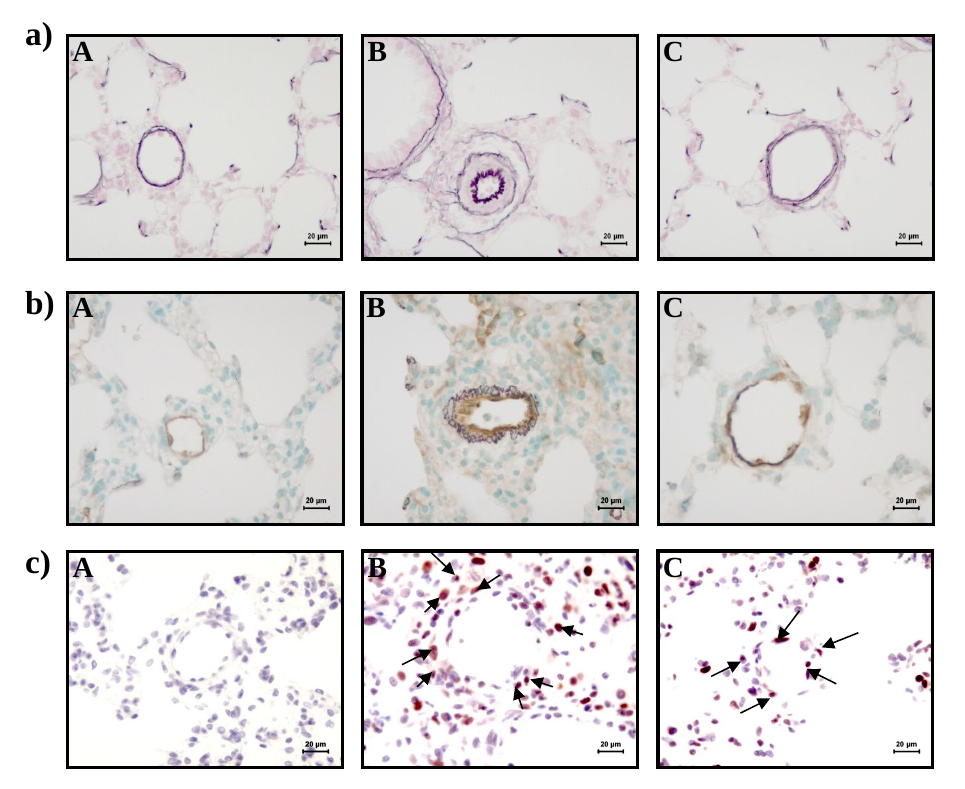

a)
A
B
C
b)
A
B
C
c)
A
B
C

Supplement: Additional file 3 — Figure S3. Representative photomicrographs from elastica staining and immunostainings. (a) elastica staining and (b) double immunostaining (anti-α-smooth muscle actin (purple/violet color) and anti-von Willebrand factor (brown color) antibodies) were performed to assess medial wall thickness and degree of muscularization, the 2 well-known measures of pulmonary vascular remodelling. Immunostaining with anti-proliferating cell nuclear antigen (PCNA) antibody (c) was performed to assess the in situ proliferation state of pulmonary vascular cells. Arrows indicate the PCNA-positive nuclei/cells. The representative photographs from stained lung tissues of different experimental groups are shown (A - healthy controls, B - monocrotaline (MCT)-placebo and C - MCT-TBC3711). [file 1465-9921-12-87-S3.PPT]

## Slide 1
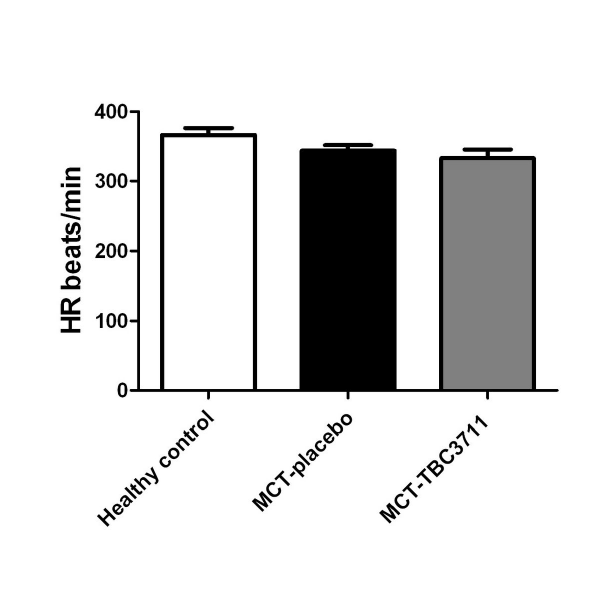

Supplement: Additional file 4 — Figure S4. Effect of TBC3711 on heart rate (HR). The heart rate (beats/min) was measured by echocardiography, as described in Methods and values of different experimental groups are given. [file 1465-9921-12-87-S4.PPT]
